# Characterization and Functional Analysis of Trim38 in the Immune Response of the Large Yellow Croaker (*Larimichthys crocea*) Against *Pseudomonas plecoglossicida* Infection

**DOI:** 10.3390/ijms26094150

**Published:** 2025-04-27

**Authors:** Qiaoying Li, Hongling Wu, Ying Huang, Dinaer Yekefenhazi, Wenzheng Zou, Fang Han

**Affiliations:** State Key Laboratory of Mariculture Breeding, Fisheries College, Jimei University, Xiamen 361000, China; 17601168505@163.com (Q.L.); 18878468726@163.com (H.W.); 202211908023@jmu.com (Y.H.); s2745005@ed.ac.uk (D.Y.)

**Keywords:** Trim38, large yellow croaker, *Pseudomonas plecoglossicida*, innate immunity, transcriptome, NF-κB signaling

## Abstract

The large yellow croaker (*Larimichthys crocea*) is a cornerstone species in Chinese marine aquaculture, yet bacterial infections—particularly visceral white nodules disease (VWND) caused by *Pseudomonas plecoglossicida*—severely compromise its production. This study aimed to elucidate the immunoregulatory mechanisms of tripartite motif-containing protein 38 in the large yellow croaker (*Lctrim38*) during bacterial infections, with an emphasis on host–pathogen interactions involving *P. plecoglossicida*, to evaluate its potential for disease-resistant breeding applications. The full-length cDNA of *Lctrim38* was cloned and characterized, with structural analysis revealing a conserved domain architecture comprising RING, B-box, coiled-coil, and PRY-SPRY motifs. Functional characterization through *Lctrim38* overexpression in large yellow croaker kidney cells (PCK cells) demonstrated significant modulation of key immune-related pathways, including TGF-β, PI3K-Akt, IL-17, and PPAR. Notably, *Lctrim38*-mediated inhibition of NF-κB signaling was shown to downregulate pro-inflammatory cytokines (TNF-α, IL-6, IFN-γ), establishing its role as a negative regulator of inflammatory responses. These findings provide insights into the immune mechanisms of Trim38 in large yellow croakers and highlight its potential as a molecular target for disease resistance breeding. Future research should explore its broader functions, including its antiviral potential.

## 1. Introduction

The innate immune system serves as the primary defense mechanism in vertebrates against pathogenic invasion, a feature of paramount importance in aquatic organisms inhabiting microbially complex environments [1]. This evolutionarily conserved system detects pathogen-associated molecular patterns (PAMPs) through pattern recognition receptors (PRRs), initiating rapid immune cascades critical for combating bacterial and viral pathogens [2]. While adaptive immunity contributes to immunological memory, teleost species predominantly rely on innate immune mechanisms to orchestrate broad-spectrum responses against aquatic pathogens [1]. In fish, immunocompetent tissues such as the head kidney (serving hematopoietic and phagocytic functions), spleen (coordinating humoral immunity), and thymus (governing T-cell maturation) form the cornerstone of cellular immunity. Mucosal surfaces—including gills, intestine, and skin—further reinforce defense through mucosa-associated lymphoid tissue (MALT), collectively constituting a sophisticated network for pathogen surveillance and elimination [3]. Recent advances have elucidated critical genes and signaling pathways underlying innate immunity, with the tripartite motif (TRIM) protein family emerging as central regulators of antiviral and antibacterial defense mechanisms [4,5].

TRIM proteins constitute a multifunctional group characterized by conserved RBCC domains (RING, B-box, and coiled-coil), orchestrating diverse biological processes ranging from intracellular signaling to immune regulation [6,7,8]. Their E3 ubiquitin ligase activity, mediated through the RING domain, enables precise modulation of key immune pathways, including NF-κB and MAPK cascades [9,10]. Ubiquitination-mediated control of these pathways regulates inflammatory responses while maintaining cellular homeostasis—a critical balance in pathogen defense [11].

TRIM family proteins have been extensively studied in antiviral immunity. Several members of the TRIM family enhance the host’s resistance to viral infections by regulating the type I interferon (IFN-I) signaling pathway [4]. For instance, Trim5 restricts HIV replication in primates [12], while Trim25 promotes the ubiquitination of RIG-I, modulating the IFN-I signaling pathway to inhibit various viral infections. Similarly, Trim22 limits viral infection by reducing the expression of viral proteins. Trim38 is another prominent member of this family, especially in mammalian models, where it plays a significant antiviral role. By ubiquitinating key signaling molecules such as TRIF and MAVS in the Toll-like receptor (TLR) and RIG-I-like receptor (RLR) pathways, Trim38 reduces excessive production of IFN-I and pro-inflammatory cytokines, thus balancing the host immune response [13,14]. This mechanism enables the host to effectively fight viruses while preventing tissue damage caused by excessive inflammation. Trim38, in particular, has been shown to protect the host from pathological damage during infections with RNA viruses such as influenza and herpesvirus by suppressing excessive inflammation.

While TRIM proteins are best characterized for their antiviral roles, their antibacterial functions remain comparatively underexplored. In mammalian systems, Trim38 has been shown to fine-tune TLR signaling through TRIF ubiquitination, thereby suppressing NF-κB activation and preventing cytokine storms during bacterial challenge [15]. In mouse models, Trim38 has been found to negatively regulate IFN-I and pro-inflammatory cytokine production by modulating TRIF in the TLR pathway, thus preventing tissue damage caused by excessive inflammation. Similarly, Trim14 enhances macrophage phagocytosis through STING-TBK1 pathway regulation, illustrating the family’s versatile immunomodulatory capacity. These findings suggest conserved yet context-dependent regulatory mechanisms across vertebrate lineages.

The large yellow croaker (*Larimichthys crocea*), a mariculture cornerstone in China [16,17], faces severe production constraints from *Pseudomonas plecoglossicida*-induced visceral white nodule disease (VWND) [18,19]. Characterized by systemic granulomatous lesions and mortality rates exceeding 60%, this pathogen devastates coastal aquaculture economies in Fujian and Zhejiang provinces [17]. Traditional disease management strategies prove inadequate against VWND’s rapid transmission, necessitating molecular approaches to enhance host resistance.

Our previous genome-wide association study identified *Lctrim38* as a key candidate gene conferring resistance to *P. plecoglossicida* infection in *L. crocea* [20]. Although mammalian Trim38 is recognized for its antiviral functions through TLR/RLR pathway modulation [21], its role in teleost antibacterial immunity remains enigmatic. This study investigates LcTrim38’s immunoregulatory mechanisms during bacterial pathogenesis through functional overexpression and transcriptomic profiling, revealing novel insights into its multifaceted roles in piscine host defense.

## 2. Results

### 2.1. Sequence Characteristics of Lctrim38

**Figure 1:**
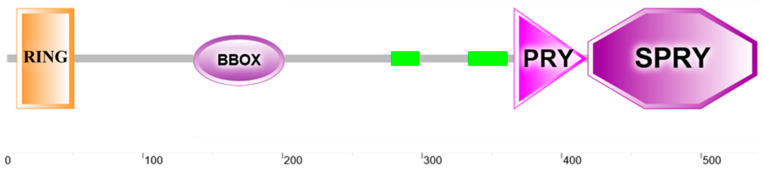
Domain architecture of LcTrim38.

**Figure 2:**
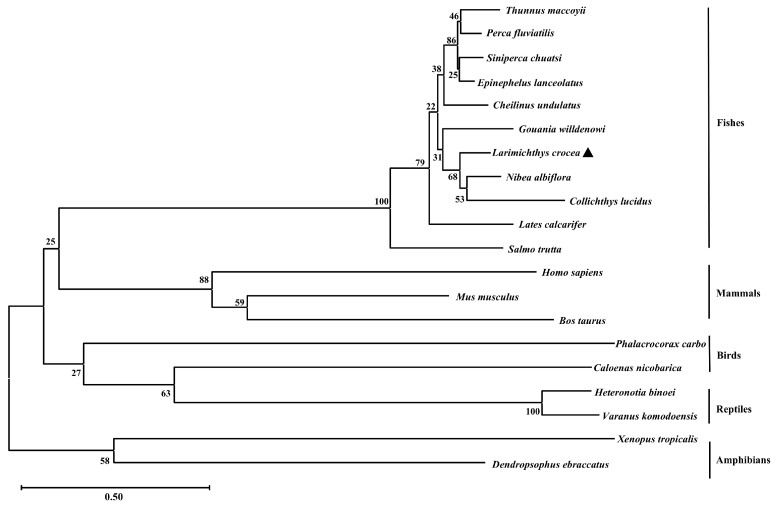
Phylogenetic analysis of LcTrim38. The black triangle indicates the *L. crocea*, the scale bar (0.50) represents genetic distance, and the GenBank accession numbers of all amino acid sequences are shown in Table 1.

The open reading frame (ORF) of *Lctrim38* was 1659 bp, encoding a protein of 552 amino acids with a predicted molecular mass of 62.85 kDa and isoelectric point of 6.19. Phosphorylation site analysis identified 58 potential modification sites in the LcTrim38 protein, comprising 36 serine (Ser), 14 threonine (Thr), and 8 tyrosine (Tyr) residues (Figure S1). Structural bioinformatic analysis revealed conserved domains characteristic of TRIM family proteins: an N-terminal RING domain (15–45 aa), followed by a B-box motif (158–198 aa), a coiled-coil domain (281–301 aa), and C-terminal PRY (369–421 aa) and SPRY (422–549 aa) domains (Figure 1). No signal peptides or transmembrane regions were predicted, consistent with its putative cytoplasmic localization. Phylogenetic reconstruction demonstrated that LcTrim38 shares closest evolutionary homology with the yellow drum (*Nibea albiflora*), followed by the spinyhead croaker (*Collichthys lucidus*), with clustering patterns aligning precisely with established taxonomic relationships among teleost species (Table 1, Figure 2).

### 2.2. Tissue Expression Pattern

**Figure 3:**
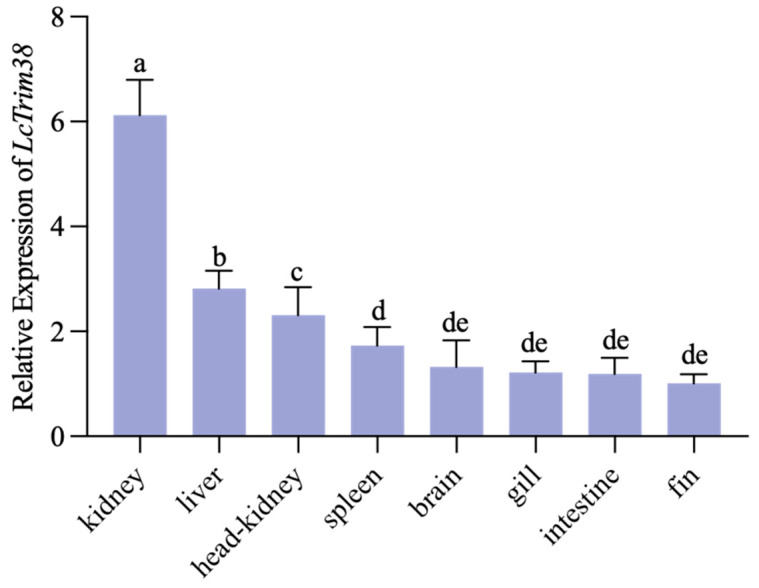
Tissue expression profile of *Lctrim38*. Expression levels of *Lctrim38* in various tissues (gill, fin, head kidney, brain, spleen, liver, intestine, and kidney) were analyzed using *β-actin* as an internal control. Lowercase letters (a–e) above the error bars indicate statistically significant differences between groups based on one-way ANOVA followed by Duncan’s multiple range test (*p* < 0.05). Groups sharing the same letter are not significantly different, while those with different letters differ significantly (*p* < 0.05).

To determine the tissue-specific expression profile of *Lctrim38*, we analyzed its expression levels in various tissues (gill, fin, head kidney, brain, spleen, liver, intestine, and kidney) using *β-actin* as the internal control. The results demonstrated that *Lctrim38* exhibited high expression levels in immune-related tissues, with the highest abundance observed in the kidney, liver, and head kidney (Figure 3).

### 2.3. Lctrim38 Expression Profiles in Response to P. plecoglossicida Infection

**Figure 4:**
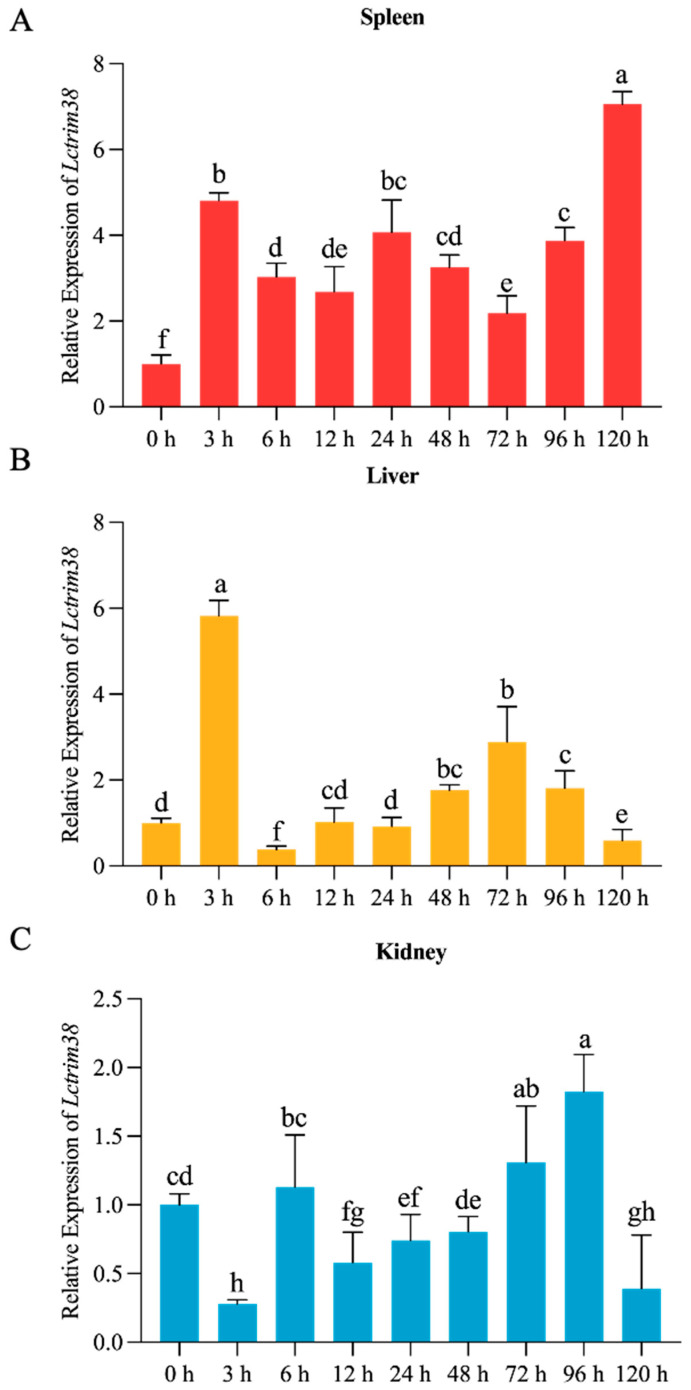
Temporal expression profile of the *Lctrim38* transcripts in spleen (**A**), liver (**B**), and kidney (**C**) at 0 h, 3 h, 6 h, 12 h, 24 h, 48 h, 72 h, 96 h, and 120 h after *P. plecoglossicida* challenge. Data are expressed as mean ± SE (n = 3). Lowercase letters (a–f) above the error bars indicate statistically significant differences between groups based on one-way ANOVA followed by Duncan’s multiple range test (*p* < 0.05). Groups sharing the same letter are not significantly different, while those with different letters differ significantly (*p* < 0.05).

To assess the impact of *P. plecoglossicida* on *Lctrim38* expression, we examined its temporal expression in the liver, spleen, and kidney at various time points: 0 h, 3 h, 6 h, 12 h, 24 h, 48 h, 72 h, 96 h, and 120 h after infection. The results are illustrated in Figure 4. Following infection, Lctrim38 expression levels in the liver and spleen increased rapidly. In the liver, expression peaked at 3 h after infection, reaching 5.7 times the control group level, then quickly declined before gradually increasing again after 12 h, peaking at approximately 0.3 times the initial value at 72 h, followed by a steady decrease. In the spleen, expression levels surged to 4.2 times the initial value at 3 h, slightly decreased thereafter, yet remained elevated at 2 to 3 times the initial value, sustaining high levels until 120 h, where it peaked at 6.8 times the baseline. In contrast, kidney expression levels significantly decreased at 3 h, returned to baseline by 6 h, and fluctuated between 0.5 to 1.3 times the initial value, peaking at 1.3 times the initial value at 96 h.

### 2.4. Transcriptome Data Analysis and Identification of DEGs

**Figure 5:**
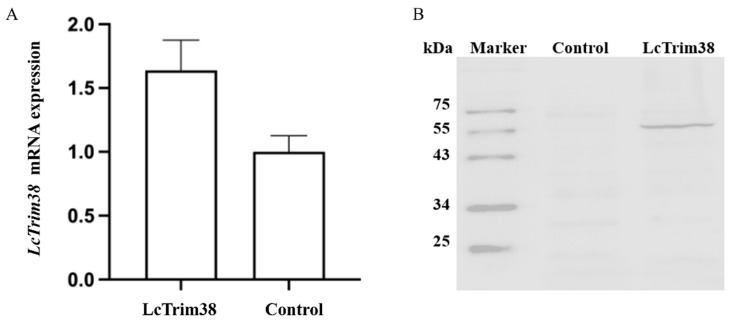
*Lctrim38* overexpression detection. (**A**) *Lctrim38* expression levels after pcDNA 3.1-*Lctrim38* and pcDNA3.1 transfection. (**B**) Western blot: “Control” means pcDNA3.1 vector was transfected into PCK cells; “LcTrim38” means overexpressed vector was transfected into PCK cells.

**Figure 6:**
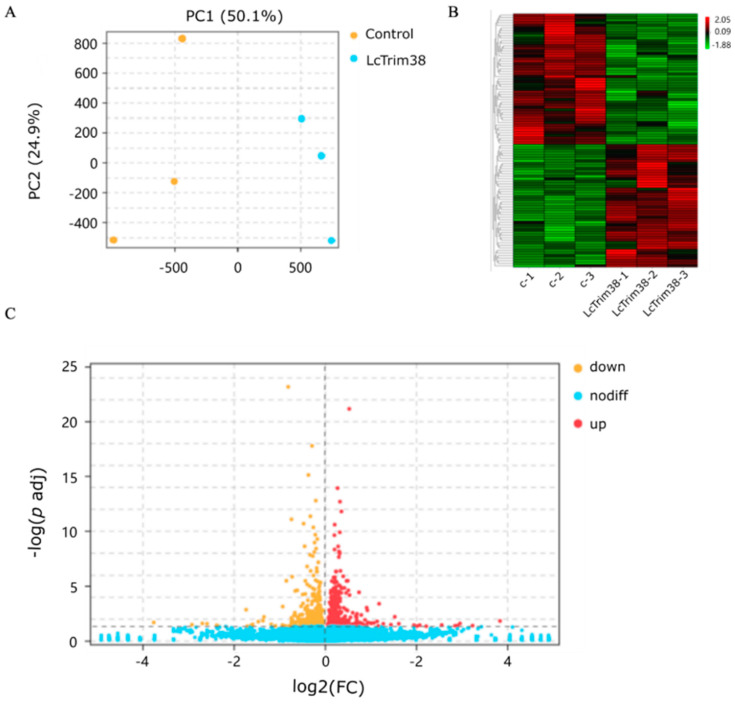
Analysis of transcriptome data and DEGs. (**A**) Principal Component Analysis. (**B**) Cluster analysis of RNA-Seq datasets using all DEGs (*p* < 0.05, log2 |(FC)| ≥ 1). “c-1, c-2, c-3” denotes control group, and “LcTrim38” denotes the experimental group subjected to overexpression. (**C**) Volcano plot illustrating DEGs in overexpressed samples.

We successfully overexpressed the Lctrim38 in PCK cells, as confirmed by RT-qPCR and WB analysis (Figure 5). To investigate the impact of Lctrim38 transfection on cellular gene expression, we conducted a transcriptome data analysis. Principal component analysis (PCA) revealed significant differentiation between the experimental group (LcTrim38-1, 2, 3) and the control group (control-1, 2, 3). Using StringTie package (https://ccb.jhu.edu/software/stringtie/, accessed on 15 January 2025), we assembled a total of 11,732 transcripts and identified 314 differentially expressed genes (DEGs) with DESeq2 package (https://support.bioconductor.org/tag/DeSeq2/, accessed on 15 January 2025), of which 160 DEGs were upregulated and 154 were downregulated (Table S1). In the volcano plot, yellow dots represent downregulated genes, red dots represent upregulated genes, and blue dots represent non-differentially expressed genes (Figure 6).

### 2.5. Enrichment Analysis of DEGs After Overexpression

**Figure 7:**
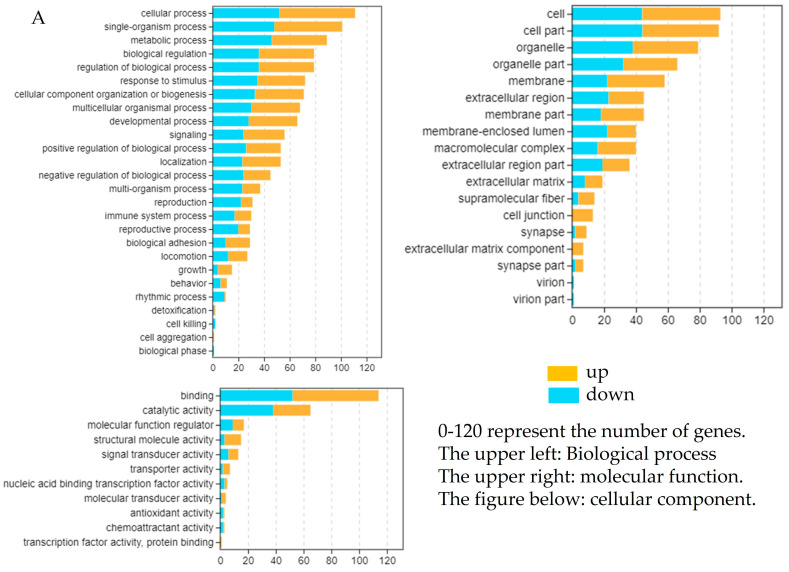

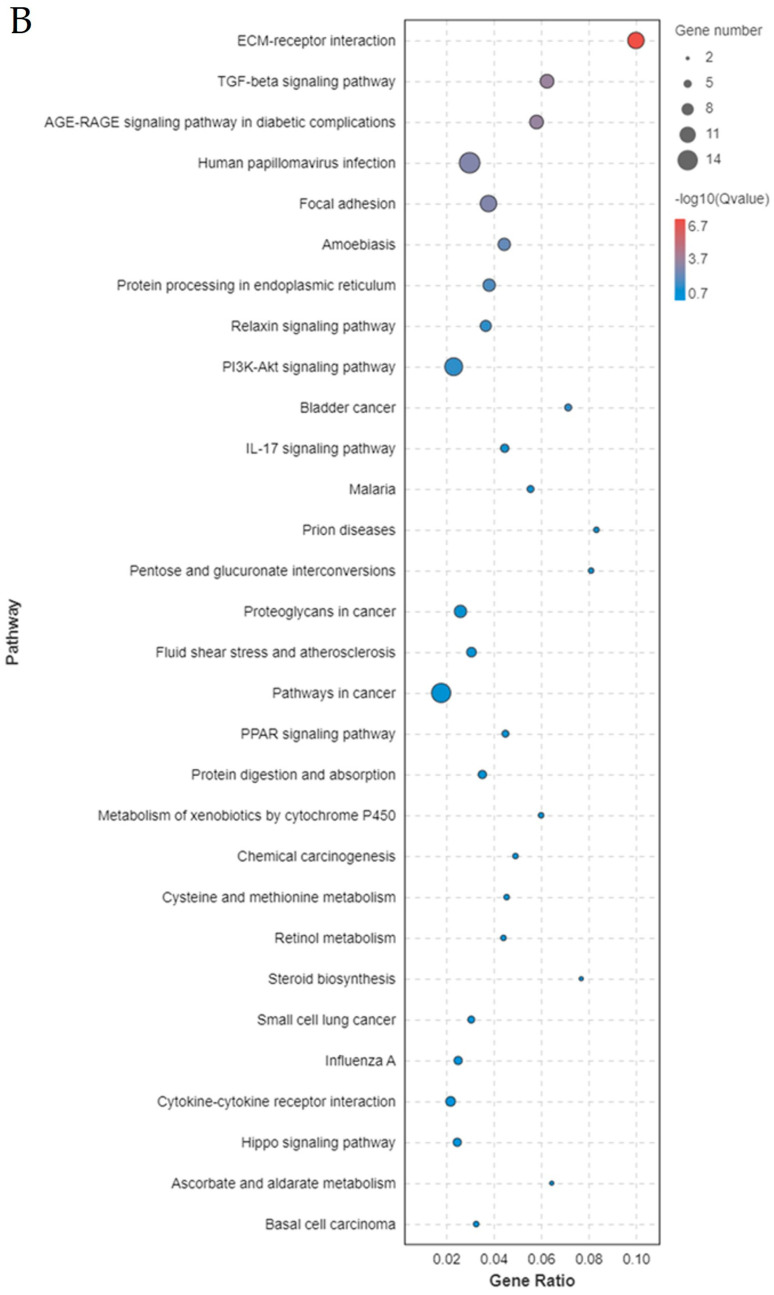
Enrichment analysis of DEGs after overexpression. (**A**) GO enrichment of DEGs after pcDNA3.1-*Lctrim38* transfection. (**B**) KEGG enrichment of DEGs after pcDNA3.1-*Lctrim38* transfection.

The GO enrichment analysis categorized the DEGs into three main classifications: cellular component (CC), biological process (BP), and molecular function (MF). Enriched pathways included regulation of the biological process, catalytic activity, transcription factor activity, protein binding, structural molecule activity, and the immune system process. The DEGs identified through RNA-seq were subjected to KEGG pathway enrichment analysis to elucidate the involvement of key signaling pathways. The significance of these pathways (TGF-β, PI3K-Akt, IL-17, and PPAR) was confirmed using the hypergeometric test with a false discovery rate (FDR) < 0.05 (Figure 7). These pathways were found to be significantly enriched among the DEGs, indicating their potential roles in the immune regulatory functions of Lctrim38.

### 2.6. Effect of Lctrim38 on Cytokine Response Triggered by LPS and Poly I:C

**Figure 8:**
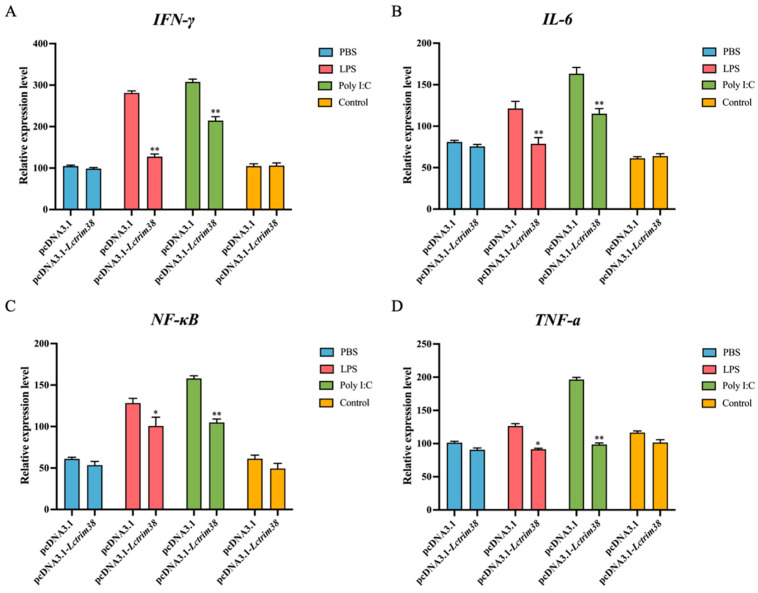
The effect of LPS and poly I:C stimulation on the expression of IFN-γ (**A**), IL-6 (**B**), NF-κB (**C**), and TNF-α (**D**) in PCK cells overexpressing *Lctrim38*. The symbols * and ** indicate significance levels of *p* < 0.05 and *p* < 0.01, respectively.

To investigate the regulatory role of *Lctrim38* in immune responses during bacterial and viral challenges, we examined the expression levels of key cytokines (IL-6, TNF-α, NF-κB, and IFN-γ) in PCK cells overexpressing Lctrim38 following stimulation with LPS (5 µg/mL) and poly I:C (10 µg/mL). The results showed that Lctrim38 significantly downregulated the expression of IL-6 and TNF-α by approximately 1.5-fold compared to the control group after stimulation with both LPS and poly I:C (*p* < 0.05). Additionally, NF-κB expression was reduced following stimulation with LPS and poly I:C (*p* < 0.05). Notably, IFN-γ expression decreased by 1.6-fold under LPS stimulation and by approximately 2-fold under poly I:C stimulation compared to the control group (*p* < 0.05) (Figure 8).

## 3. Discussion

Throughout evolutionary history, teleosts have developed sophisticated innate immune mechanisms to counter diverse pathogens, while bacterial pathogens have reciprocally evolved strategies to subvert host defenses by manipulating critical signaling pathways [22]. The TRIM protein family exemplifies this evolutionary interplay, with members exhibiting divergent functional specializations despite conserved structural frameworks. Among these, Trim38 emerges as a pivotal immunoregulator, balancing antimicrobial defense and inflammatory homeostasis through ubiquitination-mediated control of key pathways such as IFN-I signaling and cytokine production [23].

This study reveals that LcTrim38 retains conserved TRIM family architecture, featuring RING, B-box, coiled-coil, and PRY-SPRY domains—structural motifs conserved across vertebrate lineages. The presence of a functional RING domain, critical for E3 ubiquitin ligase activity [8,9], coupled with the pathogen-recognition capacity of the PRY-SPRY domain [24,25], suggests dual roles in antibacterial and antiviral immunity. Notably, evolutionary analysis reveals strong positive selection acting on teleost PRY-SPRY domains, potentially reflecting adaptations to aquatic pathogen pressures [25]. Phylogenetic conservation with *N. albiflora* and *C. lucidus* further underscores LcTrim38’s functional significance in sciaenid host defense [26].

Tissue-specific expression profiling demonstrated constitutive *Lctrim38* expression in immunologically active organs (kidney, liver, head kidney), with pronounced splenic upregulation following *P. plecoglossicida* challenge—a finding consistent with the spleen’s role as a primary infection target in visceral white spot disease [27,28]. While our 120-h observation period (exceeding standard 72–96 h protocols) revealed sustained upregulation, future studies will extend this timeframe to fully characterize late-phase expression dynamics. Moreover, Trim38 expression can be induced by TLR ligands, IFNs-I, and viral infections, further supporting its involvement in antiviral immunity [21,29]. Functional characterization via *Lctrim38* overexpression in PCK cells revealed broad immunomodulatory activity, significantly altering TGF-β, PI3K-Akt, IL-17, and PPAR pathway dynamics. These results position *Lctrim38* as a master regulator of inflammatory homeostasis, capable of suppressing pro-inflammatory cascades while maintaining immune equilibrium.

The crucial role of Trim38 during bacterial infections is reflected in its regulation of the NF-κB signaling pathway. As an essential regulator in innate immunity, NF-κB promotes the production of pro-inflammatory cytokines such as TNF-α, IL-6, and IFN-γ. Trim38 suppresses these cytokines, reducing excessive inflammation and preventing tissue damage [23]. This aligns with findings from Kim et al. [30], who reported that Trim38 reduces NF-κB activity, protecting mouse chondrocytes from inflammation-induced damage. In this study, cytokine analysis confirmed that *Lctrim38* functions as a negative regulator, with NF-κB and downstream gene expression significantly reduced following stimulation with LPS and poly I: C. Although focused on *P. plecoglossicida* (due to its severe aquaculture impact), preliminary data suggest that LcTrim38 may have broader antibacterial functions against pathogens like Vibrio and Aeromonas—a focus of ongoing research.

Furthermore, Trim38 has garnered attention for its role in antiviral immunity. Studies have shown that Trim38 acts as an interferon-stimulated gene (ISG), enhancing antiviral responses by regulating IFN-I production [21,31]. While this study focused on *Lctrim38*’s role in bacterial infections, its antiviral potential warrants further investigation. LcTrim38 modulates several signaling pathways, including TGF-β, PI3K-Akt, IL-17, and PPAR, which are critical for cell proliferation, differentiation, and immune regulation. These findings suggest that LcTrim38 not only suppresses pro-inflammatory responses but also plays a pivotal role in maintaining immune homeostasis through multiple mechanisms, highlighting its multifaceted role in host defense [32,33]. Future studies should consider using tissues from infected fish to directly assess the expression of pro-inflammatory cytokines and immune cell changes. Additionally, investigating the mechanisms by which LcTrim38 interacts with other immune-related proteins could provide further insights into its multifaceted functions in host defense. This approach would help elucidate the broader role of LcTrim38 in both antibacterial and antiviral immunity, contributing to the development of novel disease resistance strategies in aquaculture.

Notably, while cell-based experiments provided mechanistic insights, we acknowledge the need for in vivo validation. Future work will employ CRISPR-Cas9 edited fish to examine tissue-specific regulation and pathogen interactions in whole-organism contexts. These studies will be complemented by: Co-IP/mass spectrometry to identify interacting partners; structural analysis of functional domains; and multi-omics approaches to elucidate regulatory networks.

## 4. Materials and Methods

### 4.1. Experimental Fish

Healthy large yellow croakers (*Larimichthys crocea*), with an average weight of 28.5 ± 8.5 g, were obtained from Ningde Jinling Aquatic Technology Co. (Ningde, China). Healthy juveniles were reared for one week in a pathogen-free laboratory at a water temperature of 18 °C. During this acclimation period, fish were fed commercial feed twice daily at 8:00 a.m. and 6:00 p.m. Twice-daily feeding followed standard aquaculture practice. To maintain water quality, we performed the daily removal of waste/uneaten feed and 50% water changes. *P. plecoglossicida*, isolated from naturally infected fish, was used for challenge experiments. Bacterial suspensions cultured to a concentration of 1.0 × 10^9^ CFU/mL were evenly sprayed into the challenge tanks (4 m × 2 m × 0.8 m, with a water depth of 20 cm) to achieve a final concentration of 1.0 × 10^6^ CFU/mL [28], ensuring thorough contact between the bacteria and the fish. A portion of the fish were retained as a non-challenged control group, which were not subjected to bacterial challenge and served as the baseline for comparison. After a 3 h challenge period, all the challenged fish were transferred to tanks with clean, aerated seawater, maintaining the pre-challenge water temperature and salinity for continued rearing. The fish were maintained on the same feeding regimen of commercial feed twice daily at 8:00 a.m. and 6:00 p.m. throughout the experiment.

At 0 h (before challenge) and 3, 6, 12, 24, 48, 72, 96, and 120 h after challenge, six large yellow croakers were randomly selected from the challenged group, anesthetized with clove oil, and quickly dissected to collect their kidneys, spleens, and livers. These tissues were placed in RNA protection solution and stored at −80 °C for later extraction. Additionally, various tissues, including the kidney, liver, head kidney, spleen, brain, gill, intestine, and fin, were meticulously collected from three healthy fish for the purpose of profiling tissue expression. Each sample was processed and analyzed independently to ensure the accuracy and reliability of the data. To further validate the reproducibility of the results, three independent sample processing and analyses were conducted at each time point.

All animal in vivo experiments were conducted following protocols approved by the Animal Care and Use Committee at the College of Aquaculture, Jimei University.

### 4.2. RNA Extraction and Reverse Transcription

Tissue total RNA was extracted using the TransZol method (TransGen Biotech, Beijing, China), followed by an assessment of the quality and concentration. The first-strand cDNA was synthesized using the GoScript™ Reverse Transcription System (Promega, Madison, WI, USA) according to the manufacturer’s instructions.

### 4.3. Cloning and Bioinformatics Analysis of Lctrim38

The full-length cDNA sequence of *Lctrim38* was obtained from our laboratory’s transcriptome database. Specific primers were designed according to the DNA Assembly Mix Plus Kit (Beijing LABLEAD Inc., Beijing, China) (Table 2). The PCR procedure consisted of a pre-denaturation step at 95 °C for 3 min, followed by 35 amplification cycles (95 °C for 15 s, 58 °C for 40 s, 72 °C for 1 min), and a final extension at 72 °C for 5 min. Bioinformatics analysis was carried out using the method described previously [18].

### 4.4. Quantitative Real-Time PCR (RT-qPCR)

To quantitatively analyze *Lctrim38* expression, specific primer pairs were designed (Table 2), with *β-actin* serving as the internal control. The cDNA was diluted 80 times and used as the template for RT-qPCR. The RT-qPCR amplification was performed using ChamQ™ Universal SYBR qPCR Master Mix (Vazyme, Nanjing, China) in a 20 μL reaction system, with the procedure consisting of pre-denaturation at 95 °C for 30 s, denaturation at 95 °C for 10 s, annealing at 60 °C for 30 s, and extension at 72 °C for 30 s, repeated for 40 cycles. The melting curve steps were as follows: 95 °C for 15 s, 60 °C for 60 s, and 95 °C for 15 s. Relative expression levels were conducted using the 2^−ΔΔCt^ method [34].

### 4.5. Lctrim38 Overexpression

The overexpression vector pcDNA3.1-*Lctrim38* was constructed using the pcDNA3.1/myc-His A plasmid. Primer pairs were designed to match the digested sites and the vector’s sequence (Table 1). The recombinant plasmids pcDNA3.1-*Lctrim38* and pcDNA3.1 were then introduced into large yellow croaker kidney cells (PCK cells) via transient transfection using an electroporation apparatus. The PCK cell line was cultured in Leibovitz’s L-15 medium supplemented with 10% fetal bovine serum at 28 °C [35]. The presence and expression levels of pcDNA3.1-*LcTrim38* and pcDNA3.1 proteins were detected by Western blot (WB) and RT-qPCR after a 24-h incubation at 28 °C, as described in Section 4.4.

### 4.6. cDNA Library Construction and Sequencing

Total RNA was extracted from cells transfected with either the pcDNA3.1 or pcDNA3.1-*Lctrim38* recombinant plasmid using the TransZol Up Plus RNA Kit (TransGen Biotech, Beijing, China), according to the manufacturer’s instructions. The qualified RNA samples were used for library construction and high-throughput sequencing on the Illumina NovaSeq Xplus platform, performed by Novogene Co., Ltd (Beijing, China).

### 4.7. Transcriptomic Analysis

Ensuring the integrity of bioinformatics analysis necessitates the exclusion of low-quality and unidentified nucleotides from raw data. Thus, rigorous quality control is an essential preliminary step. FastQC software (version 0.12.1) was utilized to filter out error-prone sequences, yielding a set of high-quality reads. These reads were then aligned to the reference genome of *L. crocea* (https://www.ncbi.nlm.nih.gov/assembly/GCF_000972845.2#, accessed on 15 January 2025), utilizing STAR software (version 2.5.3a). The expression matrix of reads successfully aligned to both genes and exons was generated using FeatureCounts software (version 2.0.1). Differential expression analysis was conducted using DESeq2, identifying significantly differentially expressed genes (DEGs) based on the criteria of *p* adj < 0.05 and |log2(Fold Change) | ≥ 1. Additionally, Gene Ontology (GO) and Kyoto Encyclopedia of Genes and Genomes (KEGG) pathway enrichment analyses were performed.

### 4.8. Effect of Overexpressed Lctrim38 on Cytokine Expression

The PCK cell line was transfected with the pcDNA3.1-*Lctrim38* overexpression vector (or an empty vector as a control). After 24 h of transfection, the cells were stimulated with 5 µg/mL LPS and 10 µg/mL poly I:C, respectively. The control group received an equivalent volume of 1× PBS. Following thorough mixing, the cultures were incubated at 37 °C for 6 h. At the end of the stimulation period, the medium was removed from the 6-well plates, and 1 mL of 1× PBS was added to each well to gently wash away dead cells. Subsequently, 0.2 mL of trypsin was added per well, and after a 5-min incubation period, the cells were collected into 1.5 mL EP tubes. The cells were then centrifuged at 13,000 rpm for 10 min at room temperature. The supernatant was discarded, and the cell pellet was retained. RNA extraction and cDNA synthesis were performed as described in Section 2.2. The expression levels of IFN-γ, IL-6, TNF-α, and NF-κB were measured using RT-qPCR. Specific primers are listed in Table 2, and the RT-qPCR protocol adhered to the procedures outlined in Section 2.4.

The PCK cell line was transfected with the pcDNA3.1-*Lctrim38* overexpression vector (or an empty vector as a control) and stimulated with LPS (5 µg/mL) and poly I:C (10 µg/mL) for 6 h. After 24 h of transfection, the cell lines were stimulated with 5 μg/mL LPS and 10 μg/mL poly I:C, respectively. The control group received an equivalent volume of 1× PBS. Following thorough mixing, the cultures were incubated at 37 °C for 6 h. At the end of the stimulation period, the medium was removed from the 6-well plates, and 1 mL of 1× PBS was added to each well to gently wash away dead cells. Subsequently, 0.2 mL of trypsin was added per well, and after a 5-min incubation, the cells were collected into 1.5 mL EP tubes. The cells were then centrifuged at 13,000 rpm for 10 min at room temperature, the supernatant was discarded, and the cell pellet was retained. RNA extraction and cDNA synthesis were performed as described in Section 2.2. The expression levels of IFN-γ, IL-6, TNF-α, and NF-κB were measured using RT-qPCR. Specific primers are listed in Table 1, and the RT-qPCR protocol adhered to the procedures outlined in Section 2.4.

### 4.9. Statistical Analysis

Experimental data were presented as means ± standard error (SE). One-way ANOVA followed by Duncan’s multiple range test was used to determine statistical significance, with *p* < 0.05 considered significant. Analyses were conducted using SPSS software (version 28.0.1). The experimental data were analyzed using SPSS software. For the analysis of cell expression profiles and changes in expression profiles, Duncan’s multiple range test was used to determine significant differences between different treatment groups. For the analysis of changes in cytokine expression after overexpression, one-way ANOVA was used to compare differences between groups. The mean and SE were calculated directly using SPSS software for descriptive statistics.

## 5. Conclusions

This study establishes LcTrim38 as a critical immune regulator in *Larimichthys crocea*, demonstrating its role in suppressing NF-κB signaling and modulating pro-inflammatory cytokines during *Pseudomonas plecoglossicida* infection. Our findings reveal its evolutionary conservation in sciaenid immunity, tissue-specific expression patterns in key immune organs, and broad regulatory capacity across multiple signaling pathways, including TGF-β, PI3K-Akt, and IL-17. While cell-based experiments provide mechanistic insights, future work will also include in vivo models using gene-edited fish to validate its systemic functions and explore its interactions with diverse pathogens. The dual role of LcTrim38 in balancing immune homeostasis and pathogen defense highlights its potential as a molecular target for disease-resistant breeding, immunostimulant development, and health monitoring in aquaculture. By bridging fundamental immunology with practical applications, these findings pave the way for innovative strategies to enhance sustainable aquaculture practices.

## Figures and Tables

**Table 1:** Amino acid sequence homology between LcTrim38 and its orthologs from other species.

Latin Name	Foreign Name	Identity (%)	GenBank Accession
*Larimichthys crocea*	large yellow croaker	100	XP_027127989.1
*Nibea albiflora*	yellow drum	86.86	KAG8005608.1
*Collichthys lucidus*	spinyhead croaker	84.96	TKS65205.1
*Siniperca chuatsi*	aucha perch	68.63	XP_044041494.1
*Perca fluviatilis*	english perch	66.67	XP_039648391.1
*Epinephelus lanceolatus*	giant grouper	66.31	XP_033470412.1
*Thunnus maccoyii*	southern bluefin tuna	65.23	XP_042259368.1
*Lates calcarifer*	barramundi	62.54	XP_018526461.1
*Cheilinus undulatus*	humphead wrasse	57.04	XP_041636212.1
*Salmo trutta*	brown trout	47.64	XP_029597735.1
*Gouania willdenowi*	Blue tilapia	44.12	XP_028329838.1
*Homo sapiens*	human	43.48	NP_006346.1
*Varanus komodoensis*	komodo dragon	41.58	XP_044307824.1
*Bos taurus*	cattle	29.16	NP_001029746.1
*Phalacrocorax carbo*	great cormorant fairywren	28.30	XP_064330074.1
*Heteronotia binoei*	binoes gecko	28.14	XP_060095092.1
*Caloenas nicobarica*	nicobar igeon	27.98	XP_065510802.1
*Dendropsophus ebraccatus*	hourglass tree frog	27.55	XP_069838323.1
*Xenopus tropicalis*	western clawed frog	27.27	XP_031753047.1
*Mus musculus*	mouse	25.41	NP_001025106.1

**Table 2:** Primers used in this study.

Primer Name	Sequence (5′-3′)	Usage
*Lctrim38*-F	ATGGAATATCTGAGAAGTCTGCTG	ORF amplification
*Lctrim38*-R	GTGTGTTTGAGTTACAGGTGTCAT	
*Lctrim38*-qF	ACTCCAGACTCCCAACTT	
*Lctrim38*-qR	AGACCAGCGACTCTATGA	
*β-actin*-qF	TTATGAAGGCTATGCCCTGCC	
*β-actin*-qR	TGAAGGAGTAGCCACGCTCTGT	
*INF-γ*-qF	AGGTCATTCAGATGTACCGGATA	
*INF-γ*-qR	TTCCTTGATGGTCTCCACACT	RT-qPCR
*TNF-α*-qF	TAAGCAACAAGACCACCACTTC	
*TNF-α*-qR	TCTCCAGATTCCAGATGTCAGG	
*IL6*-qF	AAGCCAGAGCTGTGCAGATG	
*IL6*-qR	CTGGCATTTGTGGTTGGGTC	
*NF-κB*-qF	GAGCTCAAGATCTGCCGAGT	
*NF-κB*-qR	ATCAGCTTGCGAAAAGGAGC	
*Lctrim38*-oF	AACGGGCCCTCTAGACTCGAGATGGAATATCTGAGAAGTCTGCTGTC	overexpression
*Lctrim38*-oR	TAGTCCAGTGTGGTGGAATTCTTAAGTTACAGCTGTTATGACGAGAGG	

Note: EcoR I enzyme restriction site (GAATTC) and Xho I enzyme restriction site (CTCGAG) are underlined.

## Data Availability

The data supporting the findings of this study are available within the article. Additional datasets generated or analyzed during this study are available from the corresponding author upon reasonable request. RNA sequencing data will be deposited in the NCBI Sequence Read Archive (SRA) upon submission of the manuscript.

## Supplementary Materials

The following supporting information can be downloaded at: https://www.mdpi.com/article/10.3390/ijms26094150/s1.
