# Supplementary material for: Characterization and Functional Analysis of Trim38 in the Immune Response of the Large Yellow Croaker (Larimichthys crocea) Against Pseudomonas plecoglossicida Infection
Source: Int J Mol Sci. 2025 Apr 27;26(9):4150. doi: 10.3390/ijms26094150 (PMC12071835; doi:10.3390/ijms26094150)
Supplement: Supplementary file 1 [file ijms-26-04150-s001.zip › ijms-3512341-supplementary.pdf]

Figure S1

1 atg gaa tat ctg aga agt ctg ctg tcg gag gac ttc caa cgt tct gtc tgt ctg gat gtg

1 M E Y L R S L L S E D F Q R S V C L D V

61 ttc act gag cca gtc tca aca ccg tgt caa cac acc atc atc aat gca tgt gac att tta

21 F T E P V S T P C Q H T I I N A C D I L

121 cag tgt cca ttt tgc aga ctg ata gta tgt tgc att gtc ctt ttg ttt gtt ttt gtt ttt

41 Q C P F C R L I V C C I V L L F V F V F

181 gtt tgt ttg gtt tgt tac ttc ata ttg ata tgt tgg att gaa tac caa gac agc tca ttc

61 V C L V C Y F I L I C W I E Y Q D S S F

241 cca gct aag gta gct gct gtg cat gag aag ttt ttt caa gtc aaa tcc tcg act cca gac

81 P A K V A A V H E K F F Q V K S S T P D

301 tcc caa ctt cct gaa aca gct gac gtt ctt tgt gat atc tgc tct gag aga aag aca aag

101 S Q L P E T A D V L C D I C S E R K T K

361 gct gtt aaa tcc tgc ttg atg tgc cta gct tct ttc tgt aaa gtg cac ctt gag cca cat

121 A V K S C L M C L A S F C K V H L E P H

421 cat aga gtc gct ggt ctc aaa agc cac aca ttg tta gac cct gta aag aac ctc gat gac

141 H R V A G L K S H T L L D P V K N L D D

481 agg atg tgc aaa att cac aac aag ttg aca gaa ctg tac tgt agg aca gac aag gcc tgt

161 R M C K I H N K L T E L Y C R T D K A C

541 atc tgt gcc ttg tgt ttc aaa acc aat cac aag ggt cat aaa gtt gtc ctg ctt gag gaa

181 I C A L C F K T N H K G H K V V L L E E

601 gaa tat gaa gca gtg atg tca aaa aaa gat gca gca atg gca aat atc caa aag atg ata

201 E Y E A V M S K K D A A M A N I Q K M I

661 caa tca cgg tcc aag aag att gct gaa att gga aac tcg gtt gat aaa gct aag aaa gag

221 Q S R S K K I A E I G N S V D K A K K E

721 aaa gaa gcc agt gtg cag gtc ttc act gac ttg atc tcc tcc att cag aga tgc cag gcc

241 K E A S V Q V F T D L I S S I Q R C Q A

781 gag ctt gtt gag gtg att gag gag agg tac gca gcc aca aag caa aag gct gaa ggt ttc

261 E L V E V I E E R Y A A T K Q K A E G F

841 ctc aca gaa ctg aag atg gaa gtc acc gag ctc aaa agc aga agc agc cag ctg gag cag

281 L T E L K M E V T E L K S R S S Q L E Q

901 ctg tca cag tct gag gat cac cat cat ttt ctc cag agc ttc cca aac ttg tgt tct cct

301 L S Q S E D H H H F L Q S F P N L C S P

961 tta aac aaa gac tgg acc aac act ggt gtt cac agt gat ctg tct ttt gag gca gtg aga

321 L N K D W T N T G V H S D L S F E A V R

1021 gat gtt gta acc caa ctg aaa cac aga gtt gat gaa gta atg gaa gag ctt cct gag atc

341 D V V T Q L K H R V D E V M E E L P E I

1081 aaa ata aaa aga atg aga gga cat gca gtg gat ttg act ttt gac cct gac aca gca tat

361 K I K R M R G H A V D L T F D P D T A Y

1141 tgc tca ctt gtc ata agc cag gat gga aaa caa gtg ata gct gga gac aca gaa cag agt

381 C S L V I S Q D G K Q V I A G D T E Q S

1201 cca ccc aac aat cca aaa agg ttt gaa atg ttt aca gag gtt ttg aca aag gag ggg ttc

401 P P N N P K R F E M F T E V L T K E G F

1261 aca aca ggg aag ttt tat tat gag ctg caa gtg gaa gga agt act agg tgg gtt att ggg

```
421   T   T   G   K   F   Y   Y   E   L   Q   V   E   G   S   T   R   W   V   I   G
1321 gtg gtc aga gag tca gta aat aga aag atg gat gca cct ctg tca gtt gaa aat gga tac
441   V   V   R   E   S   V   N   R   K   M   D   A   P   L   S   V   E   N   G   Y
1381 tgg acc att ggg ctt gat gag ggt ata tat agc gca cac agg tcc acg gga caa agt gat
461   W   T   I   G   L   D   E   G   I   Y   S   A   H   R   S   T   G   Q   S   D
1441 aaa atc aca atg aaa gaa aaa ctt gag atg gtg ggc atc ttt gtg gac tat aat aag gga
481   K   I   T   M   K   E   K   L   E   M   V   G   I   F   V   D   Y   N   K   G
1501 gtg gtt tct ttc tat gat gtg att tct aaa tca cat atc tat tct ttc agt ggc tgc cac
501   V   V   S   F   Y   D   V   I   S   K   S   H   I   Y   S   F   S   G   C   H
1561 ttt aca gag aaa ctc tat cca tac ttc tac ctg aaa ccc aac ata aat gta act aac cct
521   F   T   E   K   L   Y   P   Y   F   Y   L   K   P   N   I   N   V   T   N   P
1621gcc cct ctc atc atg aca cct gta act caa aca cac tga
541   A   P   L   I   M   T   P   V   T   Q   T   H   *
```

Figure S1. Sequence analysis of *LcTrim38*.

The coding sequence and deduced amino acid sequence of *LcTrim38* are shown. Stop codons are indicated with asterisks (*). Structural domains are highlighted: RING (blue), B-Box (gray), PRY (yellow), and SPRY (green).

Table S1. Differentially expressed genes in *LcTrim38* overexpression analysis.

**Gene Name**	**log2FC**	***p adjust***
stat1	-0.7327	8.96E-12
gvin1	-0.71887	1.49E-06
gimap9	-0.84648	3.46E-06
has1	-0.60626	2.58E-05
has1	-0.65139	2.89E-05
gag-pol	0.748518	3.85E-05
trim65	1.189309	0.000429
tjp2	-8.58246	0.000658
gvin1	-0.926	0.000771
abhd15	-1.7282	0.001502
znf644	-6.90689	0.001638
psma6	0.930661	0.001708
rnf213b	-0.65821	0.00274
thsd4	0.611075	0.00281
dmpk	0.703552	0.003592
iffo1	0.798057	0.004508
mov10b.2	-0.67765	0.00491
melk	-0.64556	0.005074
tnf	0.677111	0.005915
mccc2	-1.33628	0.006847
wdsub1	0.69525	0.007167
dck	-0.5872	0.007818
gimap1	-0.73432	0.0084
x-element	7.351675	0.011154
ncf2	0.879202	0.012804
raver2	0.699629	0.012997
pik3r5	-0.71272	0.013182
uck2a	-1.35863	0.01483
p4ha3	-0.72761	0.015044
slc13a5	3.841302	0.015846
ogn	-1.46016	0.018051
tsen15	-0.64752	0.019925
sar2567	0.70502	0.020929
amigo1	0.940921	0.021699
mamdc4	-3.75489	0.021909
rnf180	-0.653	0.021977
stim2	0.768925	0.022048
pcdhgc3	0.681928	0.022194
slc22a23	1.075664	0.023969

znf703	1.015267	0.0265
acot2	2.963474	0.026915
cacna2d4	0.815392	0.027181
ccr3	-0.72698	0.028234
ppp2r5b	-2.58496	0.028273
maz	1.280753	0.028549
hmbox1	0.748174	0.028948
tmem50b	0.633176	0.030083
samd11	0.6033	0.030558
pol	-1.58496	0.032234
cmpk2	-0.64173	0.032245
vmp1	1.448358	0.034503
rab38	-2.91754	0.034868
ca1	-0.93085	0.036521
tkt	2.562936	0.037192
acss2	0.696946	0.037341
gnb3	1.952171	0.03788
fam219a	2.079727	0.038183
hsp30	-0.67288	0.038932
kifap3	-2.37504	0.039527
pro-pol	2.910448	0.041391
mafg	3.239466	0.042265
slc4a11	-5.98489	0.043159
homer2	2.855052	0.043278
sema7a	-0.86507	0.044449
brca2	-1.08597	0.044749
pank1	1.171648	0.044981
agr2	-8.49185	0.045192
tmem117	2.018859	0.046664
pisd	0.613419	0.047982
doc2b	-7.05889	0.048171
trim11	-1.70178	0.048205
gpc5	2.222392	0.04835
ift140	-0.61138	0.049393
kcnc1	5.984893	0.049528
dbnl-a	-2.32193	0.04954
mafaa	6.491853	0.049781
nudt7	-0.64361	0.049792
gng12	-0.74406	0.049961
